# Three-dimensional, patient-specific simulation of cerebral blood flow velocities as a new approach for individualized stroke prevention and treatment

**DOI:** 10.1016/j.csbj.2025.08.022

**Published:** 2025-08-21

**Authors:** Johanna Rosemarie Leyhe, Carla Marie Reinecke-Lüthge, Ala Jamous, Mathias Bähr, Sasan Kheirandish, Aria Alimi, Artjom Avakian, Ilko L. Maier

**Affiliations:** a Department of Neurology, University Medical Center Göttingen, Germany; b Institute of Diagnostic and Interventional Neuroradiology, University Medical Center Göttingen, Germany; c ELPIS Simulation GmbH, Hann. Münden, Germany

**Keywords:** Simulation, Cerebral perfusion, Stroke, Individual treatment

## Abstract

**Background:**

Three-dimensional simulations of cerebral blood flow are a highly promising approach for individualized diagnostics and therapy planning in cerebrovascular disease. The aim of this study is to validate simulated blood flow velocities generated by a patient-specific model using the innovative Stroke Quantification software (StroQ, ELPIS Simulation GmbH, Germany).

**Methods:**

In this study, consecutive patients with transient ischemic attack (TIA) or minor ischemic stroke (defined as NIHSS ≤ 4) treated at a tertiary stroke center have been investigated. The patients underwent both computed tomography angiography (CTA) and neurovascular ultrasound (nvUS) of the extra- and intracranial arteries. Patients with relatively large artery atherosclerosis (LAA) such as significant plaques or stenosis are excluded in this study. 3D-models of the brain-supplying arteries were created using standard CTA with 0.75 mm slice thickness. Blood flow of the intracranial arteries was simulated using finite volume and fluid-structure interaction methods and employing nvUS-derived flow velocities and systemic blood pressure of the extracranial internal carotid arteries (ICA) and vertebral arteries (VA). The simulated flow velocities of all intracranial arteries were then compared to the transcranial color-coded duplex sonography TCCD-derived measurements using intraclass correlation coefficients, Pearson correlations, Bland–Altman, and scatter plots.

**Results:**

Patient-specific artery models were created for 53 consecutive ischemic stroke patients without relevant LAA. The results demonstrated a high accuracy between TCCD- and simulation-derived velocities for peak systolic velocities (PSV). Intraclass correlation coefficients were moderate for three arteries and good to excellent for all remaining intracranial arteries (ICC ≥ 0.750, p < 0.001). Overall, only small differences between simulation-derived velocities and TCCD were observed (PSV: mean difference −2.6 cm/s, −4.5 %, end-diastolic velocities (EDV): mean difference 0.5 cm/s, 1.4 % and mean flow velocity (MFV): mean difference −0.3 cm/s, −3.3 %). Pearson correlations between TCCD- and simulation-derived PSV, EDV and MFV velocities were significant for all intracranial arteries (p < 0.001).

**Conclusions:**

The study revealed accurate flow velocity values derived from the patient-specific artery simulation of the intracranial arteries as compared to the clinical gold standard represented by TCCD. Simulation of cerebral perfusion may support personalized medicine by helping to tailor blood pressure targets and guide decisions on invasive reperfusion strategies (e.g., carotid endarterectomy or stenting), based on individual vascular anatomy and physiological parameters.

## Highlights

• 3D simulations of cerebral blood flow offer a promising approach to personalizing treatment for cerebrovascular diseases.
• We found highly accurate flow velocity values derived by the patient-specific simulation compared to the TCCD gold standard.
• Cerebral perfusion simulation may help tailor blood pressure targets and guide decisions on invasive reperfusion strategies.

## Graphical Abstract

**Figure:**
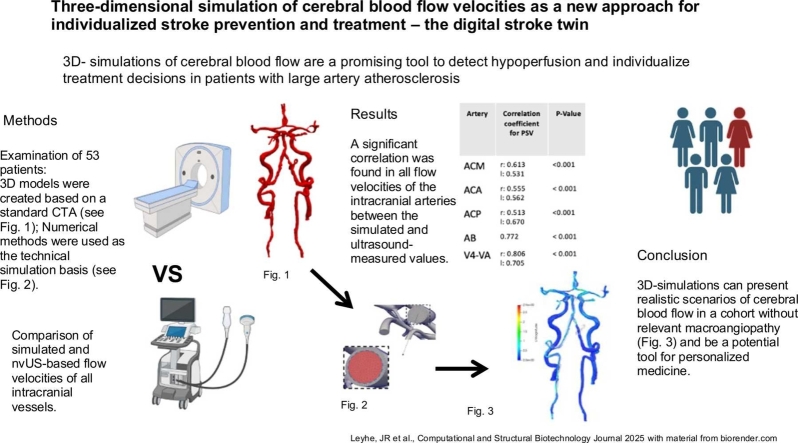

## 1 Introduction

Stroke is a leading cause of death and disability worldwide with increasing incidence rates across all age groups [1], [2]. Therefore, effective prevention and treatment approaches are crucial to reduce both the global and individual burden of stroke. Consequently, there is a growing need for more individualized tools to optimize the prevention and treatment of stroke. Anatomical variations of the brain-supplying arteries, most notably the completeness of the Circle of Willis (CoW), play a crucial role in determining the vulnerability and providing a potential compensation during states of cerebral hypoperfusion [3]. This assertion is predominantly valid for large artery atherosclerosis (LAA), a predominant cause of ischemic stroke [4]. The variability in vulnerability for ischemic stroke (based on the prevalence of family history, cardiovascular risk factors and the anatomy of brain-supplying vessels) remains an area of interest for neurovascular physicians worldwide since treatment recommendations for primary and secondary prevention [4] of stroke rely on findings from large clinical trials.

The selection of diagnostic modalities, such as computed tomography angiography (CTA), magnetic resonance angiography (MRA) or neurovascular ultrasound (nvUS) is based on the extent and type of information they can provide while subsequent treatment decisions are often guided by the data derived from averaged patient cohorts. Furthermore, the employed imaging modalities provide limited information on dynamic aspects of cerebral perfusion. CTA and MRA offer precise information regarding vessel morphology in a relatively expeditious manner; however, they are limited in terms of spatial resolution and lack hemodynamic data. nvUS, on the other hand, is a reliable, non-invasive measurement method to determine cerebral blood flow velocities with major limitations regarding inter-rater reliability, expertise of the investigator and image quality [5], [6]. The latter can be severely impaired in patients with insufficient transtemporal insonation windows, which has been observed in up to 20 % of cases [7], [8], [9]. Three-dimensional simulations of cerebral blood flow have been identified as a potentially valuable tool for individualized treatment decisions in patients with LAA of the brain, particularly in cases where there is a high risk of ischemic stroke. The primary approach adopted to investigate the cerebral blood flow was the zero-dimensional model, a rudimentary mathematical model employed for the analysis of blood flow within the CoW [5]. This was subsequently followed by the one-dimensional model [6]. The utilization of this model resulted in the numerical variation of the vessels of the CoW, thereby demonstrating a range of hemodynamic effects. The computational fluid dynamics of three-dimensional models were introduced to simulate the blood flow in aneurysms and intracranial stenoses [7], [8]. As indicated by the existing models, a sufficient correlation was observed between the calculated cerebral blood flow and the ultrasound measurements. Currently, there is a lack of implementation of measurements based on three-dimensional models in clinical practice and interdisciplinary research [10]. The majority of existing models are not based on real patient data and do not offer the simulation of the cerebral blood flow in a patient-specific 3D-model which are based on individual patient vessel imaging and physiological data.

The objective of the present study is to validate an innovative software (StroQ, ELPIS Simulation GmbH, Germany, https://elpissimulation.com) for three-dimensional simulations of cerebral blood flow in a cohort of consecutive stroke patients without relevant LAA. Here, we aim to compare the simulated blood flow velocities of the intracranial arteries with nvUS-derived values, while the simulation is based on the individual anatomy of the patients, the flow velocities of the extracranial arteries and other physiological parameters.

## 2 Materials and methods

This monocentric, prospective study analyzed consecutive clinical and imaging data from patients with transient ischemic attack (TIA) or minor ischemic stroke (defined as NIHSS ≤ 4) admitted to our tertiary university stroke center between January and November of 2023 were analyzed. Patients were eligible if they were > 18 years of age and had a full stroke workup including CTA and nvUS of extra- and intracranial arteries, in which relevant LAA such as hemodynamic relevant plaques or stenosis had been excluded. Also, sufficient Doppler spectra including automatically acquired envelope-curve peak systolic- (PSV), end-diastolic (EDV)- and mean flow velocities (MFV) in all extra- and intracranial arteries could reliably be determined. Transcranial and transnuchal color-coded duplex sonography (TCCD) was included for the determination of the aforementioned velocities in the middle cerebral (MCA)-, anterior cerebral (ACA)- and posterior cerebral arteries (PCA) as well as the vertebral arteries segment 4 (V4-VA) and the basilar artery (BA). CTA data helped to create 3D-models of the extracranial and intracranial brain-supplying arteries (for details, see below). The clinical data encompassed basic demographic information, medical history, blood pressure and heart rate on admission, during nvUS, and during CTA to nvUS, as well as hemoglobin level and hematocrit on admission. Data was extracted from the in-hospital documentation systems ICCA (IntelliSpace Critical Care and Anaesthesia, Philips) and ixserv (ixmid software technology GmbH).

All patients provided informed consent for the use of their clinical and imaging data. The study was approved by the ethics committee of the University Medical Center Göttingen (approval number 6/5/24).

### 2.1 CT-angiography

Single phase CTA was acquired using a 128-slice multidetector CT scanner (Siemens SOMATOM Definition AS+ or Siemens SOMATOM Definition Edge; Siemens Healthcare Sector, Forchheim, Germany; 120 kV, 120 reference mAs, 0.8 pitch, 2 × 64 × 0.6 mm collimation). Intravenous injection of 65 mL contrast agent, followed by 40 mL of saline chaser with bolus triggering in the aortic arch (100 HU threshold; 5-second delay in bolus watch for Definition AS+ and 10-second delay in bolus watch for Definition Edge; CTA started with 3-second delay after reaching threshold; acquisition volume and scan direction from carina to vertex; bv37 convolution kernel reconstruction). CTA data were then primarily reconstructed to axial images with a slice thickness of 0.75 mm, and secondary reconstructions were performed for further radiological interpretation.

### 2.2 Neurovascular ultrasound of the extra- and intracranial brain-supplying arteries

Extracranial nvUS of the ICA and V2-VA was performed using a GE Logique s8 XDclear system with brightness-mode (B-mode) imaging, pulsed duplex scanning and color Doppler flow imaging at 8–12 MHz. The procedure was carried out by a specialist with at least five years of experience and the certification from the “Deutsche Gesellschaft für Ultraschall in der Medizin (DEGUM) e.V.” The nvUS examinations were verified by a DEGUM-certified specialist with more than 10 years of nvUS experience. In case of inconsistent findings or insufficient imaging quality, the nvUS was repeated by the specialist. As part of the nvUS, TCCD was performed with the same ultrasound system using a 3–4 mHz transcranial probe.

Blood flow velocities of the extracranial vessels were measured in a longitudinal plane with fully visible vessels, ending at the cranial and caudal B-mode image. PSV, EDV and MFV were automatically determined by the nvUS system in one representative cardiac cycle. The MCA, ACA, and PCA were examined using transtemporal TCCD, contingent on the availability of sufficient transtemporal bone window. The latter was present if at least 75 % of the contralateral calotte was visible. For transnuchal TCCD, the patient was placed on the right side of the body with the chin placed on the chest. The probe was then placed at the transnuchal insonation site and the V4-VAs and BA were visualized. All velocities of the intracranial arteries were determined using angle correction if their flow direction could be determined. In cases with limited assessment of the flow direction, no angle correction had been applied.

### 2.3 Geometrical model for the simulation

**Fig. 1:**
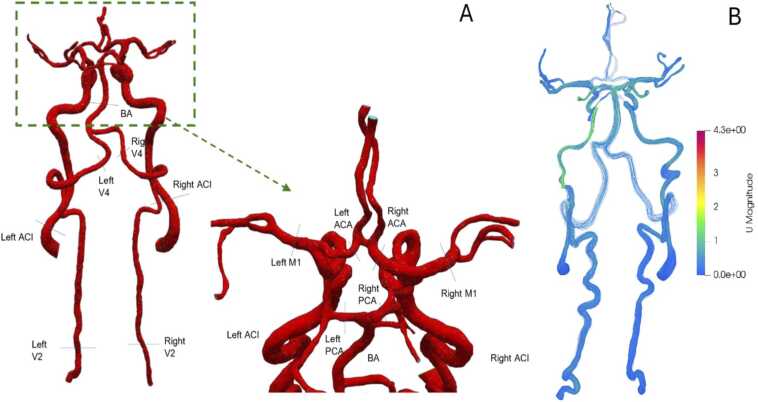
A: representative reconstruction of extracranial- and intracranial brain-supplying, arteries derived by CTA. B: representative model of blood flow simulation in a, patient with unilateral reduced diameter of the left internal carotid artery with a compensatory increase in, blood flow velocities.

We reconstructed 3D-models of the vascular geometry from DICOM images (CTA) utilizing segmentation techniques followed by a surface mesh generation (see Fig. 1). The volume rendering technique was utilized to facilitate the reconstruction of three-dimensional structures, with the DICOM images of CTA serving as the foundation. The CTA data comprised 700 slices, acquired from three distinct angles (right, anterior, and bottom), with a spatial resolution of 1 mm (see Fig. 1). The entrances of vertebral and internal carotid arteries are considered as arteries’ inlets and outlets of middle, anterior, posterior, and superior cerebral arteries as domain outlets. The skull and other non-vascular structures were eliminated during segmentation using threshold-based and region-growing algorithms. Manual correction was employed in cases of partial bone overlap.

All reconstructed models were analyzed by an experienced neuroradiologist regarding the correct anatomy of the extra- and intracranial brain-supplying vessels. If needed, segmentation was manually corrected.

### 2.4 Blood properties

The reconstructed 3D-model was used for numerical blood-flow simulations using StroQ software (developed by ELPIS Simulation GmbH, Germany). Blood was modeled as an incompressible non-Newtonian fluid with a density of 1060 $\frac{\mathrm{kg}}{m^{3}}$.The Casson model was utilized to consider the non-Newtonian behavior of the blood. The blood viscosity as a function of shear rate ($\dot{\gamma }$) [4], [10], [11] can be demonstrated following [12], [13], [14]

(1) $$\mu_{\mathrm{eff}}\left(\dot{\gamma }\right)={\left(\sqrt{\eta_{c}}+\sqrt{\tau_{c}/\dot{\gamma }}\right)}^{2},$$

where $\eta_{c}=0.0035\mathrm{pa}.s$, $\tau_{c}=0.004\mathrm{pa}$.

### 2.5 Governing equations

The governing equations, continuity (2) and momentum (3) equations known as the Navier-Stokes equations, can be written for an incompressible and non-Newtonian fluid following

$$\nabla .\vec{V}=0,\;(2)$$

$$\rho \left(\frac{\partial \vec{V}}{\partial t}+\vec{V}.\nabla \vec{V}\right)=-\nabla P+\nabla .\left(\mu_{\mathrm{eff}}\left(\nabla \vec{V}+({\nabla \vec{V})}^{T}\right)\right),\;\left(3\right)\;$$

where $\vec{V}$ is fluid velocity vector, ρ is fluid density, P is pressure and ∇ is the gradient operator.

### 2.6 Numerical method

For numerical blood flow simulations, StroQ solver was used for the numerical blood flow simulations which solves a pressure equation over time to ensure conservation of mass and momentum. This solver enforces mass conservation by solving a pressure correction equation at each time step, followed by an explicit velocity correction to satisfy the momentum equations. It optionally starts each time step by solving the momentum equation which is called a momentum predictor. With the benefit of accurate and robust simulations with large time steps, this solver can solve incompressible and transient flow simulations. The solver utilizes multiple corrector loops for numerical stability to handle high Courant number flows.

### 2.7 Boundary conditions

The wall of the arteries is considered a rigid wall in all simulation cases and no-slip condition is applied to them. The velocity boundary condition is applied to the inlet of the arteries (left and right vertebral and internal carotid arteries) which is measured by Doppler sonography (as described above). An outlet pressure boundary condition is adopted for the outlets of the arteries. The simulation of blood flow in the MCA, ACA, PCA, V4 segments of VA segments and BA were carried out using blood pressure at the time of nvUS examination and nvUS-measurements of the distal ICA and VAs in their V2 segments. Only the anatomy of the CoW was included in the simulations as a representation of primary collateral circulation. No secondary collaterals, such as leptomeningeal or pial arteries, were segmented or modeled due to the limitation of the spatial resolution of the CTA. The StroQ software (ELPIS Simulation GmbH, Germany) uses finite volume methods and multiple corrector loops to solve the Navier-Stokes equations for incompressible, non-Newtonian flow. Patient-specific inlet velocities and pressure values were applied as boundary conditions. All simulations were performed under steady-state assumptions using rigid vessel walls.

### 2.8 Statistical analysis

The statistical analyses were performed using SPSS (IBM SPSS Statistics (Version 29.0), Armonk, NY, USA, 2022). Baseline characteristics were summarized using means and standard deviations for normally distributed data or medians and interquartile ranges for non-normally distributed data. Normal distribution was tested by Kolmogorov-Smirnov and Shapiro-Wilk-test. Mean PSV, EDV and MFV of TCCD and simulation were calculated for the left and right side of an artery. Correlations between flow velocities measured by TCCD and simulation results from StroQ software were evaluated using Pearson correlations and intraclass correlation coefficients (ICCs). ICCs were interpreted according to Koo and Li [15]. P-values below 0.05 were considered statistically significant. Additionally, comparison of mean peak systolic velocities of both sides between TCCD and StroQ were conducted using Bland–Altman plots and scatter plots. Due to the lack of comparable data from similar studies, a power analysis to determine the required sample size could not be conducted.

## 3 Results

Fifty-three consecutive stroke patients with a mean age of 62.4 ± 15.2 years were included in the study (see Table 1). Twenty-four of them had a transient ischemic attack (45.2 %) and twenty-nine had a minor stroke (54.7 %). Mean systolic blood pressure (SBP) was hypertensive on admission but normalized at the time of the nvUS. Mean hemoglobin and hematocrit values were within the normal range; 12 (22.6 %) patients had anemia on admission. Regarding cerebrovascular comorbidities, dyslipidemia was the most common in 47 % of cases followed by arterial hypertension in 45 %-, prediabetic metabolic state in 13 %-, diabetes mellitus type II in 17 %- and coronary heart disease in 7 % of the cases. The mean inlet flow velocities of the ICA and VA were within normal ranges. Median time difference between CTA and nvUS was 34 ± 29.5 h (SD).

**Table 1:** Baseline characteristics of consecutive patients with minor ischemic stroke or transient ischemic attack without evidence for large vessel disease.

Variable	n = 53
Age (mean ± SD)	62.4 ± 15.2
Sex (n male, %)	40 (75.5)
Sex (n female, %)	13 (24.5)
Height (m)	1.75 ± 0.1
Body weight (kg)	81.7 ± 15.6
BMI (kg/m²)	26.4 ± 4.0
SBP (mean mmHg ± SD on admission)	147.4 ± 28.0
DBP (mean mmHg ± SD on admission)	87.3 ± 19.5
MBP (mean mmHg ± SD on admission)	107.4 ± 21.1
SBP (mean mmHg ± SD before nvUS)	133.7 ± 21.0
DBP (mean mmHg ± SD before nvUS)	80.3 ± 14.7
MBP (mean mmHg ± SD before nvUS)	98.1 ± 15.2
Hb (median, IQR g/dl on admission)	13.7; 12.5–15.2
Hk (mean % ± SD on admission)	40.6 ± 5.4
Anemia on admission (n, %)	12 (22.6)
Arterial hypertension (n, %)	29 (54.7)
Dyslipidemia (n, %)	25 (47.2)
Arterial fibrillation (n, %)	5 (9.4)
Diabetes mellitus Typ 2 (n, %)	9 (17.0)
Prediabetic metabolic state (n, %)	7 (13.2)
Coronary heart disease (n, %)	4 (7.5)
Smoking (n, %)	16 (30.2)
Harmful alcohol consumption (n, %)	3 (5.7)
PSV in extracranial ICA (mean ± cm/s)	60.1 ± 17.4
PSV in extracranial VA (mean ± cm/s)	41.6 ± 17.5
transient ischemic attack (n, %)	24 (45.2)
Minor Stroke (n, %)	29 (54.7)

SD: standard deviation, kg: kilogram, SBP: systolic blood pressure, DBP: diastolic blood pressure, MBP: mean blood pressure, IQR: interquartile range, PSV: peak systolic velocity, ICA: internal carotid artery, VA: vertebral artery

### 3.1 Comparison of blood flow velocities derived by TCCD and simulated velocities

No significant overall difference was observed in the PSVs-, EDVs and MFVs in the anterior circulation between nvUS and the simulation, with the exception of the MFVs in the left ACA, with a difference of 3.8 cm/s (9.7 %) between both modalities. All differences in the anterior circulation were not clinically significant, with an overall difference of less than 4 cm/s [16]. With respect to the posterior circulation, the PSV of the PCAs showed no significant difference between modalities, while the PSVs of the V4-VA and BA were significantly higher in the simulation with a mean deviation between 4.6 and 9.2 cm/s. For EDV and MFV, significant variation between the simulation and TCCD was observed, which ranged from 1.3 to 5.2 cm/s. As presented in Table 2, the peak systolic velocities had good ICCs for MCA right (ICC 0.755, p < 0.001), PCA left (ICC 0.769, p < 0.001), BA (ICC 0.827, p < 0.001), V4-VA right (ICC 0.876, p < 0.001), and V4-VA left (ICC 0.758, p < 0.001). For the left MCA (ICC 0.695, p < 0.001), right ACA (ICC 0.686, p < 0.001) and left (ICC 0.703, p < 0.001), and right PCA (ICC 0.661, p < 0.001) correlation coefficients were moderate. The overall difference of all intracranial brain-supplying arteries was 2.6 cm/s (4.5 %) for the PSV, 0.5 cm/s (1.4 %) for the EDV and 0.3 cm/s (3.3 %) for the MFV.Table 2.

**Table 2:** Comparison of transcranial color-coded duplex sonography (TCCD) and Stroke Quantification Software (StroQ) derived peak systolic-, end-diastolic- and mean flow velocities of the intracranial brain-supplying arteries.

	Correlation coefficient*	95 % CI (confidence interval)	p-value**	Mean Simulation (cm/s, ± SD)	Mean TCCD (cm/s ± SD)	Mean Difference (cm/s, %)	
PSV							
MCA right	0.755	0.575–0.859	< 0.001	98.6 ± 25	96.8 ± 30	−1.8; −1.8	
MCA left	0.695	0.471–0.824	< 0.001	96.3 ± 25.8	93.7 ± 26.6	−2.6; −2.7	
ACA right	0.686	0.448–0.821	< 0.001	78.8 ± 26.6	77.4 ± 18.2	−1.4; −1.8	
ACA left	0.703	0.484–0.829	< 0.001	76.0 ± 27.3	77.3 ± 20.4	1.3; 1.7	
PCA right	0.661	0.415–0.805	< 0.001	55.5 ± 17.7	58.8 ± 14.2	3.3; 5.9	
PCA left	0.769	0.592–0.869	< 0.001	58.2 ± 23.9	57.3 ± 16.1	−0.9; −1.5	
BA	0.827	0.579–0.916	< 0.001	57.8 ± 19.1	50.2 ± 16.7	−7.6; −13.1	
V4-VA right	0.876	0.757–0.924	< 0.001	51.3 ± 17.4	46.7 ± 16.9	−4.6; −9.0	
V4-VA left	0.758	0.369–0.888	< 0.001	51.4 ± 17.3	42.2 ± 15.9	- 9.2; −17.9	
Overall difference PSV:						−2.6; −4.5	
EDV							
MCA right	0.822	0.690–0.897	< 0.001	37.2 ± 12.6	37.5 ± 14.1	0.25; 0.7	
MCA left	0.820	0.686–0.897	< 0.001	36.4 ± 12.5	37 ± 13.6	0.6; 1.6	
ACA right	0.811	0.671–0.892	< 0.001	27.7 ± 11.3	29 ± 9.2	1.4; 5	
ACA left	0.712	0.502–0.834	< 0.001	26.9 ± 12	29.6 ± 9.7	2.7; 10	
PCA right	0.793	0.584–0.890	< 0.001	21.1 ± 8.7	24.4 ± 8	3.2; 15	
PCA left	0.729	0.525–0.846	< 0.001	20 ± 10.1	22.3 ± 7.9	2.3; 11.5	
BA	0.915	0.832–0.954	< 0.001	21 ± 10	18.9 ± 8.6	−2.1; −10	
V4-VA right	0.896	0.817–0.941	< 0.001	18.8 ± 8.8	17.5 ± 7.9	−1.3; −6.9	
V4-VA left	0.877	0.716–0.939	< 0.001	19.9 ± 9	17 ± 8.1	−2.9; −14.5	
Overall difference EDV:						0.5; 1.4	
MFV							
MCA right	0.651	0.392–0.799	< 0.001	33.5 ± 9.5	33.4 ± 10.7	−0.07; −0.2	
MCA left	0.726	0.524–0.842	< 0.001	33.6 ± 9.7	34.1 ± 11.2	0.5; 1.4	
ACA right	0.761	0.580–0.864	< 0.001	26.2 ± 10.1	27.5 ± 7.9	1.4; 5.3	
ACA left	0.622	0.329–0.787	< 0.001	23,8 ± 10	27.5 ± 8.5	3.8; 9.7	
PCA right	0.652	0.397–0.800	< 0.001	20.3 ± 8	22.2 ± 6.3	1.9; 9	
PCA left	0.676	0.433–0.816	< 0.001	20.2 ± 8.2	21.5 ± 6.6	1.3; 6.4	
BA	0.856	0. 687–0.927	< 0.001	20.3 ± 8.6	17.5 ± 7.2	−2.7; −13	
V4-VA right	0.771	0. 480–0.887	< 0.001	17.5 ± 8	14.2 ± 5.8	−3.3; −19	
V4-VA left	0.724	0.065–0.889	< 0.001	18.6 ± 7.7	13.4 ± 6.4	−5.2; −29	
Overall difference MFV:						−0.3; −3.3	
Overall difference of all velocities:						−0.8; −2.1	

TCCD: transcranial color-coded duplex sonography: neurovascular PSV: peak systolic velocity, EDV: end-diastolic velocity, MFV: mean flow velocity, MCA: medial cerebral artery, ACA: anterior cerebral artery, PCA: posterior cerebral artery, BA: basilar artery, V4-VA: vertebral artery in its V4 segment, SD: standard deviation; *intraclass correlation coefficient (two-way mixed-effect model, absolute agreement), **F-test with true value 0

**Fig. 2:**
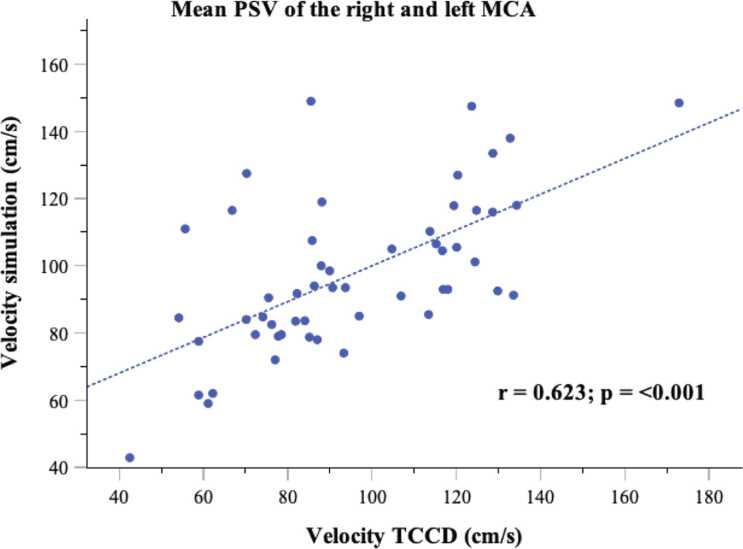
Scatter plot of the mean peak systolic velocities (PSV) in the right and left MCA, demonstrating the relationship between simulation-derived flow velocities and transcranial color-coded duplex sonography measurements (TCCD). Correlation coefficient and p-value were calculated by Pearson correlation.

Fig. 2 shows the scatter plot of the mean PSV of the right and left MCA, comparing the simulation results (variable A) with the TCCD (variable B). The data points follow an approximately linear distribution, with the points grouped relatively closely around an imaginary regression line. The data indicates a positive correlation between higher simulation velocities and higher ultrasound-measured velocities. This indicates a moderate to good correlation between the two measurement methods. The correlation coefficient r = 0.6 supports this observation and indicates a statistically relevant correlation (p < 0.001). The narrow distribution of data points indicates that the correlation is relatively stable but could still be influenced by other factors. The scatter plots of the right and left ACA, right and left PCA, right and left V4-VA and the BA showed similar results (see supplementary material).

**Fig. 3:**
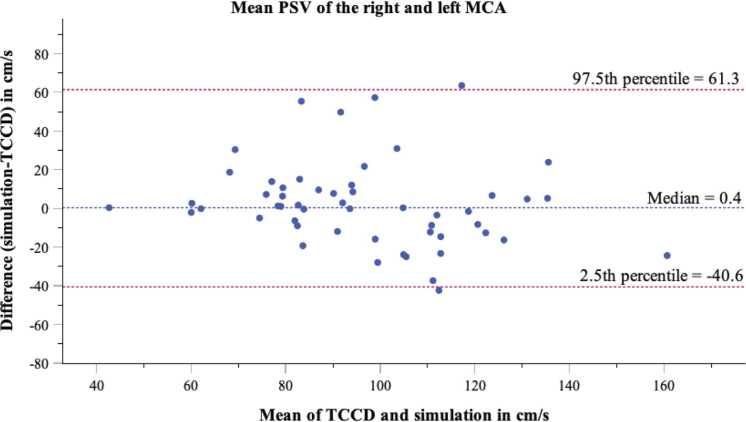
Bland–Altman plot of mean PSV in the right and left MCA of simulation and transcranial color-coded duplex sonography (TCCD). The blue dotted line shows the median, while red dotted lines represent limits of agreement at the 2.5th and 97.5th percentiles, as the differences between simulation and TCCD were not normally distributed.).

Bland–Altman analysis was conducted using the median and the 2.5th and 97.5th percentiles for limits of agreement, as the differences of the mean PSV of the right and left MCA between simulation and TCCD were not normally distributed. The median differences between simulation and TCCD is 0.4 cm/s for the PSV of the right and left MCA, which indicates a slight systematic difference; the simulation measures higher median flow velocities than the neurovascular ultrasound (see Fig. 3). The 2.5th and 97.5th percentiles, defined as percentile limits of agreement for the differences, are 61.31 cm/s and −40.6 cm/s. This means that 95 % of the pairs of measured values lie within this range. From a clinical point of view, the observed deviations are within a tolerable range, so that both methods can be regarded as largely interchangeable. Bland–Altman plots for the right and left ACA showed similar results with a median difference of 0.5 cm/s. For the right and left PCA, the median difference was −0.5 cm/s. For the BA, the median difference was 4.8 cm/s, and for the right and left V4-VA, it was 6.4 cm/s (see supplementary material).

### 3.2 Correlations between blood flow velocities derived by TCCD and simulated velocities

We found consistent and statistically significant correlations in all velocities comparing the simulation-derived results (Table 3, p < 0.001). The highest correlation coefficients were observed in the posterior circulation (BA and V4-VAs with correlation coefficients >0.7). Concerning the type of velocity result (PSV, EDV and MFV), there was no overall difference concerning the degree of positive correlation.

**Table 3:** Correlations of blood flow velocities of the intracranial brain-supplying arteries between transcranial color-coded duplex sonography (TCCD) and simulation using the Stroke Quantification software (StroQ) (n = 53).

	Correlation coefficient	p-value
PSV		
MCA right	0.613	< 0.001
MCA left	0.531	< 0.001
ACA right	0.555	< 0.001
ACA left	0.562	< 0.001
PCA right	0.513	< 0.001
PCA left	0.670	< 0.001
BA	0.772	< 0.001
V4 right	0.806	< 0.001
V4 left	0.705	< 0.001
EDV		
MCA right	0.698	< 0.001
MCA left	0.694	< 0.001
ACA right	0.699	< 0.001
ACA left	0.587	< 0.001
PCA right	0.705	< 0.001
PCA left	0.605	< 0.001
BA	0.872	< 0.001
V4 right	0.823	< 0.001
V4 left	0.827	< 0.001
MFV		
MCA right	0.481	< 0.001
MCA left	0.571	< 0.001
ACA right	0.636	< 0.001
ACA left	0.490	< 0.001
PCA right	0.511	< 0.001
PCA left	0.526	< 0.001
BA	0.803	< 0.001
V4 right	0.734	< 0.001
V4 left	0.732	< 0.001

StroQ: Stroke Quantification software, PSV: peak systolic velocity, EDV: end-diastolic velocity, MFV: mean flow velocity, MCA: medial cerebral artery, ACA: anterior cerebral artery, PCA: posterior cerebral artery, BA: basilar artery, V4-VA: Vertebral artery in its V4 segment

### 3.3 Flow volume derived by the simulation with StroQ

The flow volumes of the intracranial arteries determined by StroQ are shown in supplementary table 1. As the flow volumes of the intracranial brain-supplying arteries (including intracranial ICA, MCA, ACA, PCA, V4-VA and BA) cannot be reliably determined by nvUS or TCCD, these values were compared with the published data from the literature. In comparison, all flow volumes of the intracranial arteries were within the range of measurements reported in the literature.

### 3.4 The impact of angle correction by neurovascular ultrasound

The comparison of measurements of intracranial brain-supplying arteries with and without angle correction by neurovascular ultrasound is presented in supplementary Table 2. For the right MCA, angle correction was performed in 71.7 % (38 cases) and for the left MCA in 64.2 % (34 cases) of all 53 cases. The measurement of the basilar artery was performed in 13.2 % (7 cases) with angle correction and in 86.8 % (46 cases) without any correction. The V4-segment of the right vertebral artery was measured with angle correction in 34.6 % (18 cases), and on the left side in 28.3 % (15 cases) of all cases.

Peak systolic velocities from the TCCD and simulation were compared. The differences in cases with angle correction was found to be 1.9 cm/s (2 %) for the right MCA, for the left MCA 1.9 cm/s (2 %), −4.4 cm/s (-6.5 %) for the BA, −3.5 cm/s (-6.3 %) for the right vertebral artery segment 4 and −7.9 cm/s (-12.3 %) for the left side. Measurement without angle correction showed a difference for the right MCA of −11 cm/s (-12.7 %), for the left MCA of −10.8 cm/s (-12.1 %), for the BA of −8.1 cm/s (-14.4 %), for the right vertebral artery segment 4 of −5.2 cm/s (-10.7 %) and for the left side of −9.8 cm/s (-21.1 %). There was no significant difference observed when comparing measurements with angle correction of all brain-supplying arteries. Absent of angle correction, all differences in the anterior circulation and in the PSV of the right vertebral artery were not statistically significant, while a significant difference was identified for the basilar artery and the left vertebral artery. These results were analogous to the previously mentioned differences between flow velocities derived by neurovascular ultrasound and simulation with StroQ (see Table 2).

## 4 Discussion

Three-dimensional vessel imaging represents a non-invasive methodology for the estimation of cerebral blood flow velocity. In the context of clinical decision-making, the use of simulations can facilitate the individualization of stroke therapy. By providing a realistic representation of cerebral blood flow and the ability to simulate certain scenarios, such as varying degrees of stenosis in the internal carotid artery and their impact on cerebral blood flow, numerical models can be employed to inform medical decisions regarding treatment options. In this study, we found a clinically convincing agreement between simulation-derived blood-flow velocities and those measured by TCCD as the gold standard ultrasound.

In the present study, a positive correlation between peak systolic velocities derived by the StroQ-simulation and measured by TCCD was found for all large intracranial vessels. In comparison, a difference with higher simulation-derived velocities was observed for the vertebrobasilar system in terms of the standard deviation of TCCD and simulation data. This discrepancy can be attributed to the challenging nature of angle correction in TCCD, which was observed in some cases. Without such correction, larger angles result in an underestimation of the actual blood flow velocity [17]. A subsequent comparison of the mean difference in measurements performed with and without angle correction revealed no statistically significant findings for the cases performed with angle correction (see supplementary table 2). Previous studies have also demonstrated a high degree of correlation between velocity values obtained from 3D-simulations and those derived from transcranial Doppler measurements [18]. Additionally, comparisons have been made between these velocity values and those obtained from other established measurement techniques, such as magnetic resonance imaging (MRI) scans [19]. The work of other research groups differs primarily in their boundary conditions for blood and in the material model used for the arterial walls [20], [21], [22]. The "StroQ" approach employs an innovative strategy for cerebral blood flow simulation. In comparison to other study designs, our approach involves simulating blood flow in rigid arteries, based on the assumption of incompressible and non-compliant walls as applied in the present model. Additionally, we incorporated patient-specific anatomical variations of the CoW (primary collaterals). Furthermore, we utilized individualized patient data, including blood pressure, ultrasound measurements, and blood viscosity, in the numerical calculations. In contrast to the present imaging techniques of vessel imaging, blood flow analysis, and stenosis gradient assessment, a virtual simulation does not necessitate the use of arterial or venous puncture, contrast media, or radiation. Furthermore, these examination techniques only present a snapshot of brain hemodynamics and are unable to assess additional target parameters, such as the necessary perfusion pressure in critically undersupplied tissue, the direction of flow, or its profile.

The simulation and modulation of cerebral perfusion has the potential to serve as a valuable tool for individualized blood pressure goals and the guidance of decisions regarding invasive reperfusion strategies (e.g., carotid endarterectomy or stenting). This objective could be accomplished by giving due consideration to patient-specific vessel anatomy, physiological parameters, and measurement data, including cerebral perfusion levels both prior to and following the treatment (e.g., carotid endarterectomy or stenting) of a stenosis, as well as blood pressure modifications before and afterwards. In addition, three-dimensional simulation models have the capacity to illustrate the optimal individual blood pressure range for a given individual. This enables the detection of critical undersupply of the brain, for example due to low blood pressure, and the simulation of the potential risk of a sudden blood pressure drop. In the event of asymptomatic stenosis of the intracranial cerebral arteries, a patient-specific artery model may offer comprehensive insights into cerebral perfusion and the individual risk of stroke in the event of progressive atherosclerosis in a specific vessel. Furthermore, the influence of collaterals or the simulation of post-intervention blood flow prior to the actual treatment could also be conducted. A broader knowledge of the patient's individual perfusion profile could inform treatment options that extend beyond the grade of stenosis alone [22], [23]. In addition, the implementation of intensified conservative prevention strategies could be considered. A deeper understanding of individual atherosclerosis progression could facilitate the provision of more personalized prevention strategies. For instance, it could assist in identifying those at elevated risk of stroke due to vessel variations and altered cerebral hemodynamics, which could be considered as individual biomarkers [22].

Another potential application of a patient-specific artery model is the real-time monitoring of cerebral perfusion in patients undergoing treatment on a stroke unit. By implementing individualized monitoring, a personalized treatment plan can be devised that accounts for variations in blood pressure and its impact on cerebral perfusion. This allows for the determination of optimal blood pressure ranges, which can reduce the influence of blood pressure modification on the risk of cerebral hypoperfusion (stroke risk) and the risk of cerebral hemorrhage. Other studies indicate the potential utility of 3D-simulation in emergency stroke scenarios. With regard to an individual penumbra-to-core transformation in acute ischemic stroke, 3D-simulation may offer a valuable adjunct to perfusion-based patient selection, thereby expanding the range of treatment options for patients outside the current treatment time windows [19]. Computational fluid dynamics were employed to assess the morphology of intracranial aneurysms and to categorize the risk of rupture [24], [25]. Additionally, the impact of wall shear stress as a significant risk factor for aneurysm rupture was elucidated. Another crucial objective for 3D-simulation could be to simulate wall shear stress as a biomarker for estimating atherogenesis. To further expand the applications of three-dimensional simulations, future studies should incorporate the modulation of diverse pathologies, including intra- and extracranial stenosis.

## 5 Limitations and future studies

A potential limitation of the study is that nvUS was performed during clinical routine by different examiners, which may have resulted in variability in quality. Additionally, CTA exhibited a constrained spatial resolution of 1 mm, thereby constraining the reconstruction to the initial and secondary branches of the middle cerebral artery. Secondly, manual or semi-manual corrections for the vessel segmentation and simulation were required, which is a time-consuming process that introduces the potential for inter-rater variability in this study. Notably, the current computational processing time for 3D-simulation is not conducive to real-time clinical use. As artificial intelligence gains traction in clinical practice, there may be opportunities to automate and perform these manual corrections in real time [19], [26]. Future studies could implement a more standardized system of performing nvUS and segmentation process to avoid possible variability and enable real-time-analysis. Also, the influence of arterial wall compliance should be considered in further studies. While the model allows for patient-specific simulations based on anatomical and hemodynamic input, it does not yet incorporate dynamic changes in vascular resistance or autoregulatory responses. Therefore, individual variations in vascular reactivity due to medications, endothelial dysfunction, or sympathetic tone are not accounted for. Another potential source of confounding is the lack of consideration of disease-specific vascular mechanics caused by vascular comorbidities such as hypertension and dyslipidemia. Their potential effect on vessel compliance and autoregulation was not explicitly modeled. Due to the aforementioned limited resolution of the CTA, small vessels such as penetrating arterioles, resistance arteries, or pial collaterals could not be reliably reconstructed or simulated. As such, the model does not capture microvascular perfusion, which limits its current utility in mimicking stroke therapies dependent on distal collateral recruitment or resistance modulation.” These aspects represent important future directions and should be acknowledged as limitations of the current model.

Furthermore, both CTA and neurovascular ultrasound were performed while patients were in a supine position. Cerebral hemodynamics are influenced by body posture, and changes in perfusion due to orthostatic variation have not been evaluated. It is important to note that the generalizability of the outcomes is limited to upright conditions. To date, studies have been conducted that suggest a discrepancy in blood flow velocities. However, the impact on patient outcomes remains to be elucidated [27], [28].

Due to the lack of comparable analyses, we were unable to conduct a power analysis to determine the required sample size previously. Larger and multicentric validation of the presented technique is necessary to underline the reliability of the simulated patient data.

## 6 Conclusion

Three-dimensional simulations of cerebral blood flow appear to be a viable method for presenting realistic scenarios in a patient cohort without significant macroangiopathy, as compared to ultrasound measurements. To further expand the clinical utility of a patient-specific artery model, future studies should incorporate patients with chronic cerebrovascular diseases, such as high-grade stenosis of the internal carotid artery, for the purpose of evaluating individualized revascularization therapy plans. Similarly, in patients with intracranial hemorrhage, these models could support the estimation of individualized blood pressure goals and the assessment of wall shear stress. Multi-center trials and validation against clinical outcomes will be essential to ensure generalizability, improve accuracy, and support integration into routine clinical decision-making.

## CRediT authorship contribution statement

**Maier Ilko:** Writing – review & editing, Writing – original draft, Visualization, Validation, Supervision, Resources, Project administration, Investigation, Formal analysis, Data curation, Conceptualization. **Artjom Avakian:** Writing – review & editing, Visualization, Supervision, Resources, Project administration, Conceptualization. **Johanna Rosemarie Leyhe:** Writing – original draft, Visualization, Validation, Investigation, Formal analysis, Data curation. **Carla Marie Reinecke-Lüthge:** Writing – review & editing, Visualization, Validation, Investigation, Formal analysis, Data curation. **Sasan Kheirandish:** Writing – review & editing, Visualization, Validation, Software, Methodology, Data curation. **Aria Alimi:** Writing – review & editing, Writing – original draft, Visualization, Validation, Software, Resources, Methodology, Data curation, Conceptualization. **Ala Jamous:** Writing – review & editing, Validation, Resources. **Mathias Bähr:** Writing – review & editing, Validation, Resources.

## Declaration of Competing Interest

ILM, AJ, MB, JRL and CMRL have no interest to declare. AAv and AAl are Co-founders of ELPIS simulation GmBh and SK is employed at ELPIS simulation GmBh. AAv was awarded a grant from the “Niedersächsisches Ministerium für Wirtschaft, Verkehr, Bauen und Digitalisierung“ as part of the „Hightech-Inkubatoren“ program for the 2022–2024 period. „We acknowledge support by the Open Access Publication Funds of the Göttingen University.“
