# Supplementary material for: Three-dimensional, patient-specific simulation of cerebral blood flow velocities as a new approach for individualized stroke prevention and treatment
Source: Comput Struct Biotechnol J. 2025 Aug 21;28:321–9. doi: 10.1016/j.csbj.2025.08.022 (PMC12419103; doi:10.1016/j.csbj.2025.08.022)
Supplement: Supplementary file 1 — Supplementary material [file mmc1.docx]

**Supplementary materials**

| 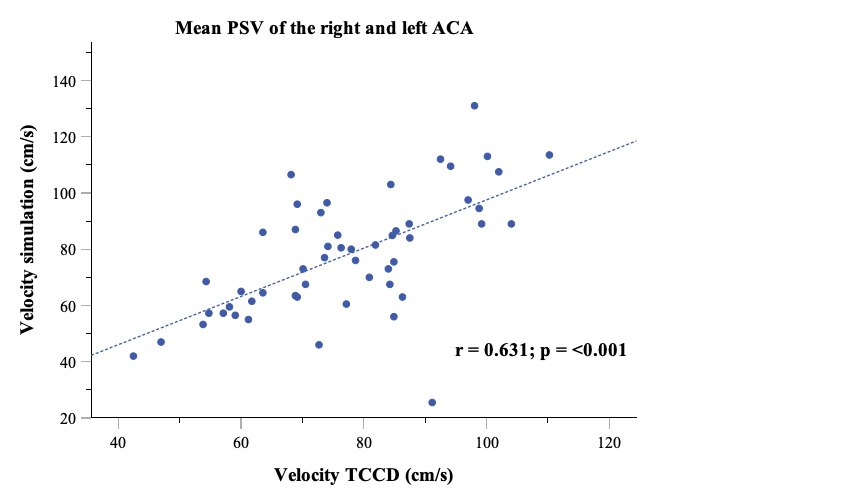  Supplementary figure 1: Scatter plot of the mean peak systolic velocities (PSV) of the right and left ACA to demonstrate the relationship between simulation-derived flow velocities and transcranial color-coded duplexsonography measurements (TCCD). Correlation coefficient and p-value were calculated by Pearson correlation.  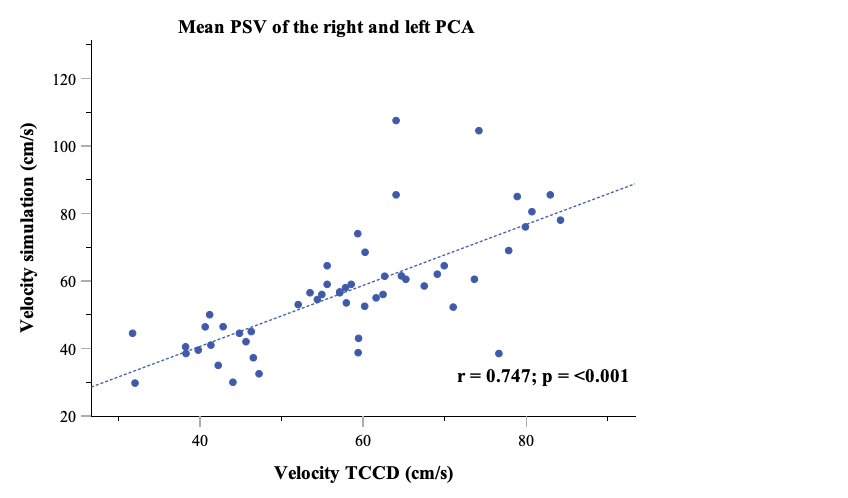  Supplementary figure 2: Scatter plot of the mean peak systolic velocities (PSV) of the right and left PCA.  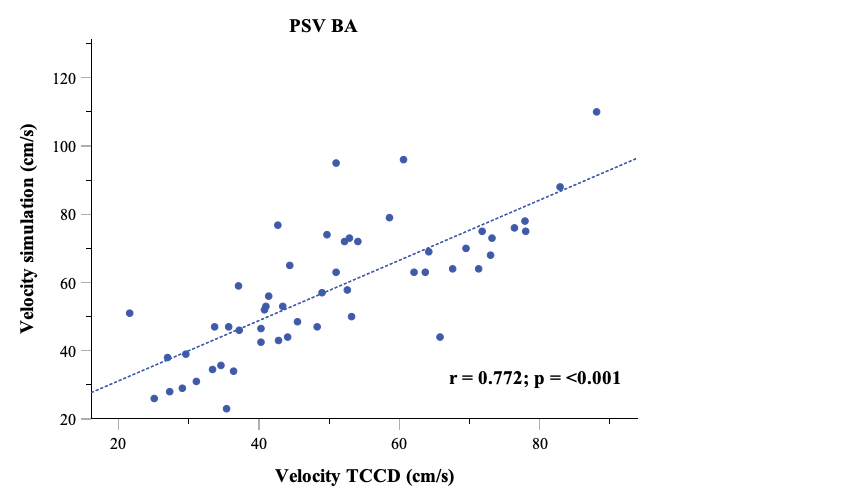  Supplementary figure 3: Scatter plot of the mean peak systolic velocities (PSV) of the BA.  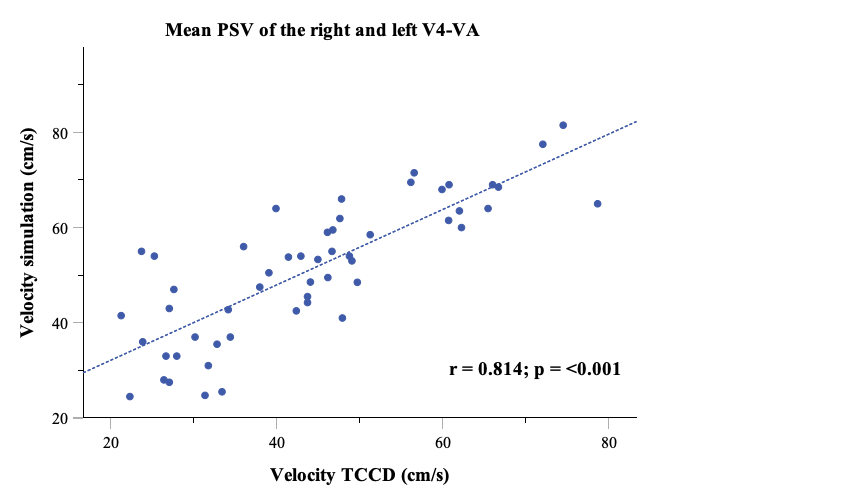  Supplementary figure 4: Scatter plot of the mean peak systolic velocities (PSV) of the right and left V4-VA.  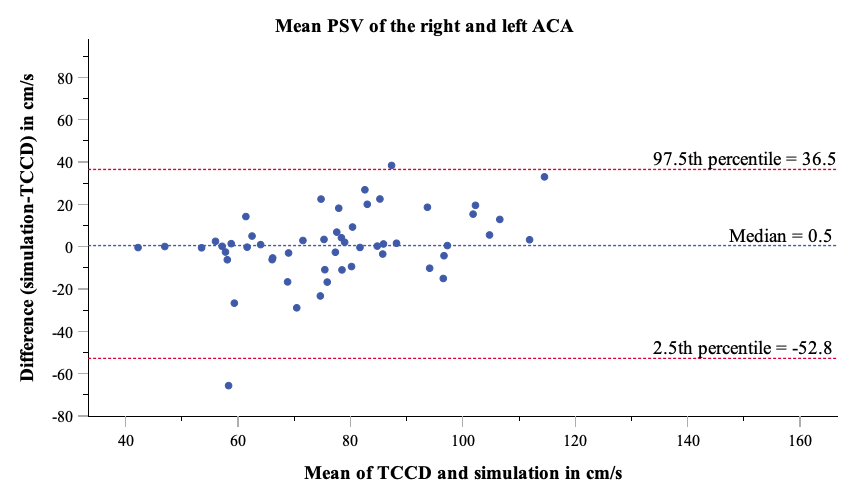  Supplementary figure 5: Bland-Altman plot of the mean PSV of the right and left ACA of simulation and transcranial color-coded duplex sonography (TCCD). The blue dotted line shows the median, while red dotted lines represent limits of agreement at the 2.5^th^ and 97.5^th^ percentiles, as the differences between simulation and TCCD were not normally distributed.  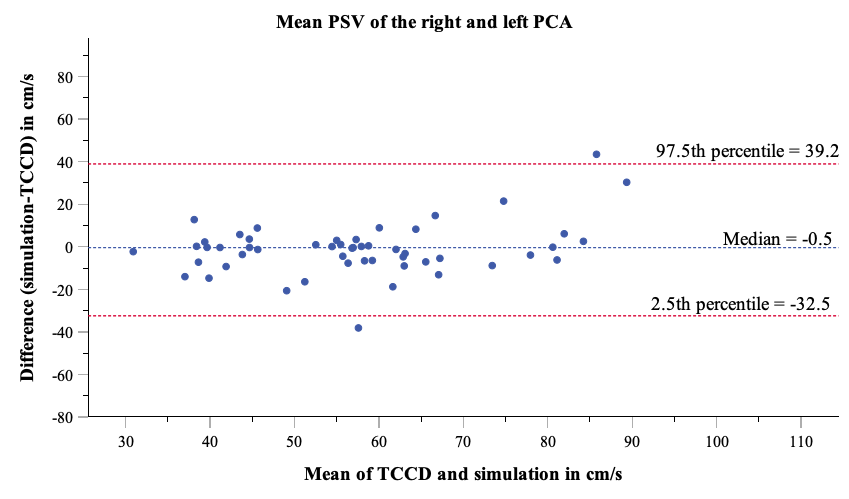  Supplementary figure 6: Bland-Altman plot of the mean PSV of the right and left PCA of simulation and transcranial color-coded duplex sonography (TCCD).  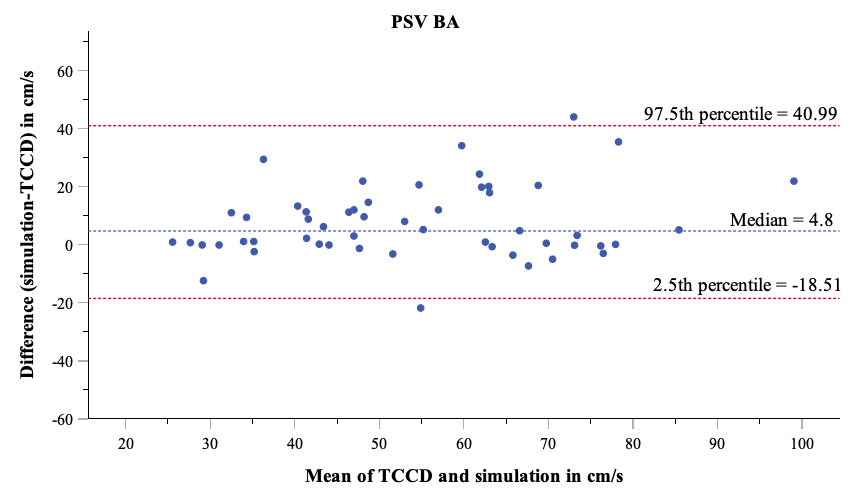  Supplementary figure 7: Bland-Altman plot of the mean PSV of the BA of simulation and transcranial color-coded duplex sonography (TCCD).  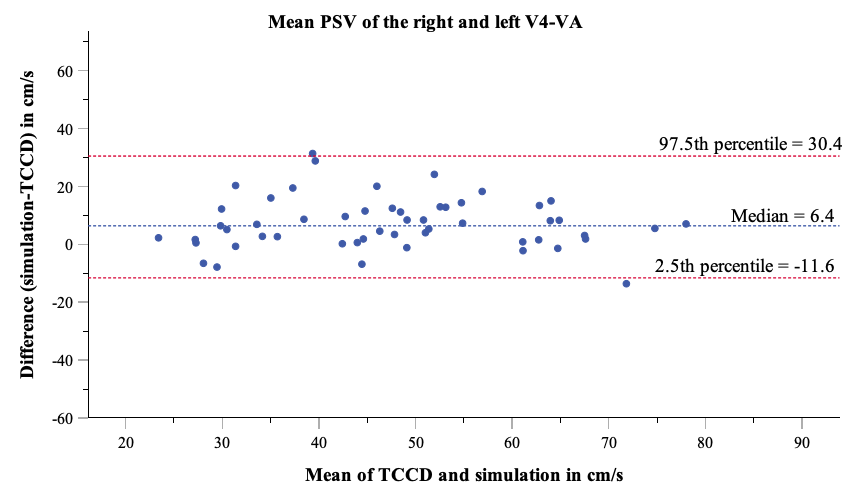  Supplementary figure 8: Bland-Altman plot of the mean PSV of the right and left V4-VA of simulation and transcranial color-coded duplex sonography (TCCD).  Supplementary table A: Systolic flow volumes as determined by Stroke Quantification Software (StroQ) in stroke patients without relevant large artery atherosclerosis (n=53) | | | | | | |  |  |  |  |  |
| --- | --- | --- | --- | --- | --- | --- | --- | --- | --- | --- | --- |
|  |  | | |  |  | |  |  |  |  |  |
|  | Flow volume | | | |  | |  |  |  |  |  |
|  | StroQ (ml/s ± SD) | | | Literature (ml/s) | Reference | |  |  |  |  |  |
| Right ICA | 5.89±2.24 | | | 5-10 | Steinman et al., 2002[24] | |  |  |  |  |  |
| Left ICA | 6.11±2.34 | | |  |  |  |  |  |  |  |  |
| Right MCA | 3.87±1.64 | | | 2-6 | Bozkurt et al. 2023[25] | |  |  |  |  |  |
| Left MCA | 3.73±1.34 | | |  |  |  |  |  |  |  |  |
| Right ACA | 1.58±0.85 | | | 1-3 |  |  |  |  |  |  |  |
| Left ACA | 1.73±1.19 | | |  |  |  |  |  |  |  |  |
| Right PCA | 0.98±0.49 | | | 0.5-2 | Wake-Buck et al. 2012[26] | |  |  |  |  |  |
| Left PCA | 1,08±0.63 | | |  |  |  |  |  |  |  |  |
| Right V4-VA | 1.6±1.32 | | | 1.5-3 |  |  |  |  |  |  |  |
| Left  V4-VA | 2.32±1.39 | | |  |  |  |  |  |  |  |  |
| BA | 3.37±1.23 | | | 3-7 |  |  |  |  |  |  |  |
| StroQ: Stroke Quantification Software, SD: standard deviation, ICA: internal carotid artery, MCA: medial cerebral artery, ACA: anterior cerebral artery, PCA: posterior cerebral artery, V4-VA: vertebral artery in its V4 segment, BA: basilar artery. | | | | | | |  |  |  |  |  |
|  |  | | |  |  | |  |  |  |  |  |
|  |  | | |  |  | |  |  |  |  |  |
|  |  | | |  |  | |  |  |  |  |  |
|  | | | | | | | | | | | |
|  | | |  | | | |  | |  | |  |
|  | |  | | | |  | |  | |  | |
|  | |  | | | |  | |  | |  | |
|  | |  | | | |  | |  | |  | |

| Supplementary table B: Comparison of measurements of the intracranial brain-supplying arteries with vs. without angle correction by neurovascular ultrasound | |  |  |  |  |  |  |  |  |  |
| --- | --- | --- | --- | --- | --- | --- | --- | --- | --- | --- |
|  | nvUS **with** angle correction (n, %) | nvUS **without** angle correction (n, %) | nvUS **with** angle correction | nvUS **without** angle correction | Simulation of those measured **with** angle correction | Simulation of those measured **without** angle correction | Difference **with** angle correction (cm/s, %) | Difference **without** angle correction (cm/s, %) | nvUS **with** angle correction vs. simulation p-value* | nvUS **without** angle correction vs. simulation p-value* |
| PSV (cm/s ± SD) |  |  |  |  |  |  |  |  |  |  |
| MCA right | 38; 71,7 | 15; 28,3 | 105.3 ± 28.0 | 75.4 ± 23.8 | 103.4 ± 24.2 | 86.3 ± 23.7 | 1.9; 1.8 | -11; -12.7 | 0.653 | 0.052 |
| MCA left | 34; 64,2 | 19; 35,8 | 102.1 ± 25.6 | 78.8 ± 21.7 | 100.1 ± 26.7 | 89.6 ± 23.1 | 1.9; 2 | -10.8; -12.1 | 0.637 | 0.090 |
|  |  |  |  |  |  |  |  |  |  |  |
| BA | 7; 13,2 | 46; 86,8 | 63.7 ± 18.5 | 48.1 ± 15.7 | 68.1 ± 23.9 | 56.2 ± 18.1 | -4.4;-6.5 | -8.1; -14.4 | 0.462 | < 0.001 |
| V4-VA right | 18; 34,6 | 34; 65,4 | 52.3 ± 14 | 43.6 ± 17.7 | 55.9 ± 16.6 | 48.8 ± 17.6 | -3.5; -6.3 | -5.2; -10.7 | 0.062 | 0.019 |
| V4-VA left | 15; 28,3 | 38; 71,7 | 56.4 ± 16.7 | 36.6 ± 11.6 | 64.3 ± 13.9 | 46.3 ± 15.9 | -7.9; -12.3 | -9.8; -21.1 | 0.057 | <0.001 |
| nvUS: neurovascular Ultrasound, MCA: medial cerebral artery, BA: basilar artery, V4-VA: vertebral artery in its V4 segment, SD: standard deviation; *paired students t-test | | | | |  |  |  |  |  |  |
